# Cetuximab-induced esophageal ulcer: the first report in literature

**DOI:** 10.3402/ljm.v9.23723

**Published:** 2014-02-24

**Authors:** Taner Babacan, Ibrahim Halil Turkbeyler, Muhammet Sait Dag, Ismail Dilli, Ozan Balakan, Kadri Altundag

**Affiliations:** 1 Department of Oncology, School of Medicine, Hacettepe University, Ankara, Turkey; 2 Department of Internal Medicine, School of Medicine, Adiyaman University, Adiyaman, Turkey. Email: turkbeyler@mynet.com; 3 Department of Gastroenterology, School of Medicine, Gaziantep University, Gaziantep, Turkey; 4 Department of Internal Medicine, School of Medicine, Gaziantep University, Gaziantep, Turkey; 5 Department of Oncology, School of Medicine, Gaziantep University, Gaziantep, Turkey

Cetuximab, a monoclonal antibody to the extracellular domain of epidermal growth factor receptor (EGFR), is indicated for the treatment of metastatic colorectal cancers and head–neck cancers. Cetuximab is generally well tolerated but some side effects, such as skin rash, malaise, vomiting, diarrhea, hypomagnesemia, and hypersensitivity reactions, have been reported (1, 2). Esophageal ulcer in patients receiving cetuximab treatment has not been described previously. We herein report a 59-year-old man diagnosed with metastatic rectal cancer with esophageal ulcers associated with cetuximab after five cycles of treatment. To the best of our knowledge, this is the first report that shows cetuximab may induce esophageal ulcers, which may be precursor lesions to a gastrointestinal tract perforation.

A 59-year-old man with constipation and pelvic pain was admitted to the gastroenterology outpatient department. A flexible rectoscopic examination revealed a rigid and painful mass encircling the lumen of the rectum. Biopsy specimen of the rectal mass confirmed a well-differentiated adenocarcinoma of the rectum. Surgery was planned, but the patient was refused the procedure and was kept under follow-up. Three months later, the patient was referred to the emergency department with acute abdominal pain, vomiting, impaired defecation, and fever. Due to intestinal obstruction, laparotomy was performed for surgical resection, although pre-operative computed tomographic (CT) scans showed evidence of possible invasion to the urinary tract and perirectal tissue. At surgery, the tumor could barely be mobilized due to direct invasion into the surrounding tissues, and the operation ended in a simple sigmoid colostomy to relieve his condition. In order to treat rectal cancer, infusional 5-FU and oxaliplatin-based chemoradiotherapy was administered. After the chemoradiotherapy, CT scan showed partial regression on the rectum and perirectal tissues, but a metastatic mass was observed on the left surrenal. Positron emission tomography (PET)-CT scan confirmed this surrenal mass and rectal involvement with a moderate Fluorodeoxyglucose (FDG) accumulation. Because v-Ki-ras2 Kirsten rat sarcoma viral oncogene (KRAS) sequencing of a tumor biopsy sample showed wild-type, he was started on second-line chemotherapy with cetuximab 500 mg/m^2^ and irinotecan 180 mg/m^2^ every 2 weeks. After three cycles of cetuximab and irinotecan, the patient had odynophagia and endoscopy was planned but the patient refused the procedure. Chemotherapy was continued with the same protocol because the patient deteriorated after five cycles of treatment. Patient refused third-line chemotherapy setting and was followed up with the best supportive care. Two weeks later, the patient was admitted to our outpatient department with hematemesis and melena. The patient's hemoglobin level was revealed to be 6.8 g/dl. An endoscopic examination showed a large, deep, white exuding ulcer in the lower third of the esophagus. There was a visible vessel in the middle of the ulcer. Argon plasma coagulation stopped the bleeding. Proton pump inhibitor was also started and biopsies were taken from the edge of the ulcer. Pathological evaluation of the ulcer showed acute inflammation. Cytomegalovirus (CMV) IgG and IgM was also negative. There was no prior history of use of any medications known to induce esophageal ulcer. Based on these laboratory and clinical findings, we assumed that esophageal ulcer was related to cetuximab treatment.

Gastrointestinal (GI) ulcers have been described previously in 10 of 755 patients (1.3%) with colorectal cancer who were treated with chemotherapy and bevacizumab (3). Mechanisms underlying GI perforation and ulceration in patients treated with bevacizumab are unknown; however, evidence supports that vascular endothelial growth factor (VEGF) plays a major role in this process. Tarnawski (4) described cellular and molecular mechanisms of gastrointestinal ulcer healing; this process is controlled by cytokines and growth factors, including VEGF. Esophageal ulcer in patients receiving cetuximab treatment has not been described previously, and may be the precursor lesion to a gastrointestinal tract perforation. We herein report a 59-year-old man diagnosed as metastatic rectal cancer with esophageal ulcers associated with cetuximab after five cycles of treatment. Cetuximab blocks activation of receptor-related kinases, resulting in inhibition of cell growth, apoptosis, decreased VEGF and matrix metalloproteinases production (5). Reduced levels of VEGF and matrix metalloproteinases may induce esophageal ulcer in cetuximab setting patients. It is possible that cetuximab, in addition to chemotherapy, causes esophageal mucosal inflammation resulting in mucosal breaks and ulceration. Further studies are needed to explain the exact mechanism of esophageal ulcer formation related to cetuximab. This report shows the importance of elucidating upper GI symptomatology in patients receiving cetuximab and the necessity of performing early endoscopy to rule out an esophageal ulcer and impending perforation.

*Taner Babacan*
Department of Oncology
School of Medicine
Hacettepe University
Ankara, Turkey
*Ibrahim Halil Turkbeyler*
Department of Internal Medicine
School of Medicine
Adiyaman University
Adiyaman, Turkey
Email: turkbeyler@mynet.com
*Muhammet Sait Dag*
Department of Gastroenterology
School of Medicine
Gaziantep University
Gaziantep, Turkey
*Ismail Dilli*
Department of Internal Medicine
School of Medicine
Gaziantep University
Gaziantep, Turkey
*Ozan Balakan*
Department of Oncology
School of Medicine
Gaziantep University
Gaziantep, Turkey
*Kadri Altundag*
Department of Oncology
School of Medicine
Hacettepe University
Ankara, Turkey
